# Effect of SWCNT and MWCNT on the flow of micropolar hybrid nanofluid over a curved stretching surface with induced magnetic field

**DOI:** 10.1038/s41598-020-65278-5

**Published:** 2020-05-22

**Authors:** A. M. Al-Hanaya, Farrah Sajid, Nadeem Abbas, S. Nadeem

**Affiliations:** 1 Department of Mathematis, College of Science, Princess Nora bint Abdul Rahman University, Riyadh, 11564 Saudi Arabia; 2 Department of Mathematics, Quaid-I-Azam University 45320, Islamabad, 44000 Pakistan; 3 Mathematics and its Applications in Life Sciences Research Group, Ton Duc Thang University, Ho Chi Minh City, Vietnam; 4 Faculty of Mathematics and Statistics, Ton Duc Thang University, Ho Chi Minh City, Vietnam

**Keywords:** Mathematics and computing, Nanoscience and technology

## Abstract

We considered the magnetized micro polar fluid with hybrid nanomaterial flow over a curved stretching surface. We discussed the effects of single wall carbon nanotube and multiwall carbon nanotube with base fluids (water and propylene glycol). Under the flow assumptions, we developed the mathematical model and applied the boundary layer approximations to reduce the system of partial differential equations. Further, the suitable similarity transformations are applied on the partial differential equations to make dimensionless system. The dimensionless system solved by means of numerical scheme via bvp4c shooting methods. Involving the dimensionless physical parameters effects are highlighted in the form of graphs and tables. Additionally, significant physical quantities i.e. Nusselt number, Couple stress coefficient and Skin friction coefficient are also presented and evaluated numerically. These results are more important which may use in the field of engineering and industrial.

## Introduction

Heat transfer is an essential process of industrial sectors to establish transit of energy in the system. Huge demand of energy in the world has made the process of heat transfer a core task. In recent years a great attention has been gained by nanofluids because of their extensive usage in industrial technologies like paper, medical industry, pharmaceutical industry etc. Nanofluids are composed of nanoparticles (metals, oxides, carbides etc.) with a typical size of less than 100 nm which are dispersed into base fluids (oils, lubricants, ethylene glycol, polymeric solutions, biological fluids etc.). These are the dilute liquid suspensions of nanoparticles which are used to to improve the thermos-physical properties of base liquids and to improve their heat transfer characteristics because the solid particles have a larger thermal conductivity than liquids. The term nanofluids was first coined by Choi^1^ in 1995.Choi was of opinion that the nanofluids have much better thermal conductivity than base fluids. Nanofluids with carbon nanotubes dispersed in synthetic oil were developed at the forefront by Choi, Eastman^2^ in 2001.They found that copper and CNT results in elevated thermal conductivity as compared to those of their base fluid without dispersed CNTs. Some efforts in this direction are cited in^3–10^.

The gratifying results of nanofluids elicited researchers to think about the suspension of different combinations of nanoparticles in the base fluid. This extension of nanofluids gave rise to a remarkable class of fluids called ‘hybrid nanofluids’. When two or more nanoparticles are dispersed in base fluid than a hybrid nanofluid is evolved having intensified thermal conductivity than nanofluids containing a single type of nanoparticle. Many experimental studies have been conducted on this concept. Sadaf and Nadeem^11^ investigated that hybrid nanofluid Ag-$CuO$/water provided vibrant improve in heat transfer rate as compared to nanofluid. Li and Xuan^12^ studied the convective heat transfer and flow characteristics in a tube with constant heat flux at the wall experimentally. Some well written articles are cited in Refs. ^13–20^.

In accordance with the law of viscosity, fluids are characterized as Newtonian and Non Newtonian fluids. Non Newtonian fluids are those in which shears stress is the function of viscosity that is why these fluids are deprived of regular viscosity. The dairy products such as cheese and butter and biological fluids like blood, etc. with respect to the specific characteristics non Newtonian fluids have a variety of names. Some of them are Casson, micropolar, Jafferly, Oldroyd and Walter’s-B. In this article Non Newtonian micropolar fluid is considered. Micropolar fluids are the fluids with microstructures consisting of rigid particles dealing with micro rotation and dispersed in a viscous medium provided the particle deformation is pushed aside. These fluids have nonsymmetrical stress tensor along with polar fluid’s characteristics. The term micropolar was raised by Eringen *et al*.^21,22^. In his theory, he revealed that micropolar fluids are composed of dilute suspensions of rigid micro molecules having individual motion that assist the stress and body movements. Some eminent writings on this concept are highlighted as^23,24^. Ghadikolaei^25^ investigated MHD boundary layer analysis for micropolar dusty fluid containing Hybrid nanoparticles (Cu-Al_2_O_3_) over a porous medium. Micropolar fluid is interpreted through a porous medium by Cao *et al*.^13^. A recently papers have been done by researchers with different aspects of the micropolar fluids see Refs. ^8,26–30^.

It is an established fact that magnetic field plays a vital role in engineering processes, medical field (MRI, cancer diagnosis, etc.). The effect of induced magnetic field has broad range of applications nowadays. The study of magneto-hydrodynamic fluid has always been an area of keen interest for researchers. Such studies are apposite to magneto-aerodynamics, MHD boundary layer control technologies and nuclear by Koshiba *et al*.^31^. Kumari *et al*.^32^ investigated the boundary layer flow and heat transfer characteristics on stretching surface with induced magnetic field. The idea of nanoparticles with induced magnetic field for drug delivery is also well focused by many researchers^33–41^.

Motivated by the aforementioned works, the present study is conducted to examine the impact of the induced magnetic field on heat transfer of steady micropolar hybrid nanofluid flow course towards a curved stretched sheet. Up till now, no attempt has been made for this kind of flow as per the best of researcher’s knowledge. For an ample analysis, common fluid such as water and propylene glycol are taken as base fluids and single and Multiwall carbon nanotubes are considered as nano particles. The system of governing equations is formulated in curvilinear coordinates which is further simplified through similarity analysis. The whole computation is done using bvp4c method in computational software MATLAB.

## Thermo-physical hybrid nanoparticles and base fluids

The thermos-physical characteristics of micropolar hybrid nanofluid i.e. viscosity, density, thermal conductivity and volumetric heat capacity are defined by empirical relations as follows:

$${A}_{1}=\frac{{\mu }_{hnf}}{{\mu }_{f}}=\frac{1}{{(1-{\phi }_{1})}^{2.5}{(1-{\phi }_{2})}^{2.5}}+{K}_{1}$$

$${A}_{2}=\frac{{\rho }_{hnf}}{{\rho }_{f}}=\left((1-{\Phi }_{1})(1-{\Phi }_{2})+{\Phi }_{1}\frac{{\rho }_{s1}}{{\rho }_{f}}+{\Phi }_{2}\frac{{\rho }_{s2}}{{\rho }_{f}}\right)$$

$${A}_{3}=\frac{{k}_{hnf}}{{k}_{bf}}=\frac{{k}_{s2}+(s-1){k}_{bf}-(s-1){\Phi }_{2}({k}_{bf}-{k}_{s2})}{{k}_{s2}+(s-1){k}_{bf}+{\Phi }_{2}({k}_{bf}-{k}_{s2})}\,where\,\frac{{k}_{bf}}{{k}_{f}}=\frac{{k}_{s1}+(s-1){k}_{f}-(s-1){\Phi }_{1}({k}_{f}-{k}_{s1})}{{k}_{s1}+(s-1){k}_{f}+{\Phi }_{1}({k}_{f}-{k}_{s1})}$$

$${A}_{4}=\frac{{(p{c}_{p})}_{hnf}}{{(p{c}_{p})}_{f}}=\left((1-{\Phi }_{2})+\left\{1-{\Phi }_{1}+{\Phi }_{1}\frac{{(p{c}_{p})}_{s1}}{{(p{c}_{p})}_{f}}\right\}+{\Phi }_{2}\,\rho \frac{{(p{c}_{p})}_{s2}}{{(p{c}_{p})}_{f}}\right)$$

The following are experimentally determined values of the thermophysical characteristics of base fluids i.e. water and propylene glycol and nanoparticles i.e. single and Multiwall CNTs (Hone^42^, Ruoff and Lorentz^43^).

## Mathematical Formulation

**Figure 1:**
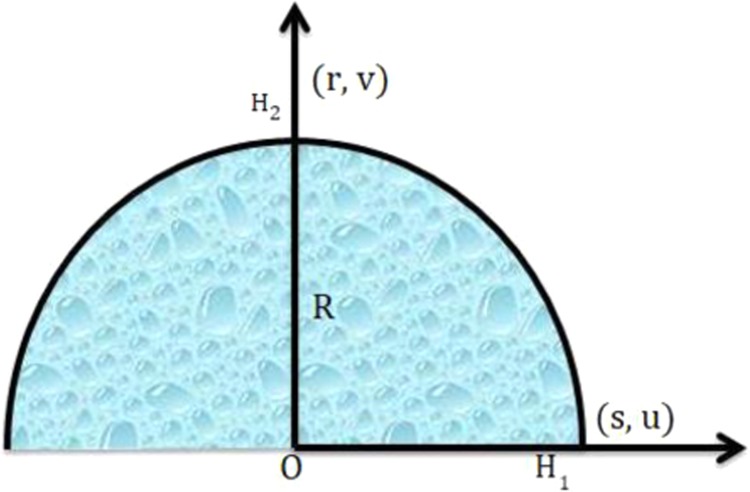
Flow of hybrid nanoparticles with base fluids.

In this investigation a steady, two-dimensional micropolar hybrid nanofluid is considered. For the mathematical description of flow, system of curvilinear coordinates is chosen and sees Fig. 1 Single and Multiwall CNTs are dispersed in water and propylene glycol taken as base fluids over a stretched sheet curved over a ring of radius R. the surface is strained along s-coordinate by applying forces of equal magnitude in the opposite direction. Let $u=as\,(a > 0)$ be the velocity of stretched surface and r-direction is orthogonal to it. Furthermore, induced magnetic field H is also introduced to discover its effect on heat transfer of the flow. Parallel and normal components of induced magnetic are ${H}_{1}\,and\,{H}_{2}$, while free stream value is taken as ${H}_{e}={H}_{0}s,\,{H}_{0}$ is the constant upstream magnetic field which equalizes ${H}_{0}$ vanishing ${H}_{2}$ at the surface. The surface temperature ${T}_{w}(s)=As/l$ is considered to be constant throughout nanofluid where A is material constant and ${T}_{w} > {T}_{\infty }$. After applying the boundary layer approximations, the continuity equation, the momentum equation, micropolar momentum equation and energy equation take the form (Refs. ^44–46^)

1 $$\frac{\partial }{\partial {\rm{r}}}(({\rm{r}}+{\rm{R}}){\rm{v}})+{\rm{R}}\frac{\partial {\rm{u}}}{\partial {\rm{s}}}=0,$$

2 $$R\frac{\partial {H}_{1}}{\partial s}+\frac{\partial }{\partial r}[(r+R){H}_{2}]=0,$$

3 $$\frac{{u}^{2}}{r+R}=\frac{1}{{\rho }_{f}}\frac{\partial p}{\partial r},$$

4 $$\frac{R}{r+R}\left(u\frac{\partial u}{\partial s}\right)+v\frac{\partial u}{\partial r}+\frac{uv}{r+R}-\frac{{\mu }_{e}}{4\pi {\rho }_{e}}\left[\frac{R}{r+R}\left({H}_{1}\frac{\partial {H}_{1}}{\partial s}\right)+{H}_{2}\frac{\partial {H}_{1}}{\partial r}+\frac{{H}_{1}{H}_{2}}{r+R}\right]=-\,\frac{1}{{\rho }_{f}}\frac{R}{r+R}\frac{\partial p}{\partial s}+(1+{K}_{1})\left(\frac{{\partial }^{2}u}{\partial {r}^{2}}+\frac{1}{r+R}\frac{\partial u}{\partial r}-\frac{u}{{(r+R)}^{2}}\right)-\frac{{k}^{\ast }}{{\rho }_{f}}\frac{\partial N}{\partial r},$$

5 $$\frac{R}{r+R}\left(u\frac{\partial {H}_{1}}{\partial s}\right)+v\frac{\partial {H}_{1}}{\partial r}+\frac{{H}_{1}{H}_{2}}{r+R}-\left[\frac{R}{r+R}\left({H}_{1}\frac{\partial u}{\partial s}\right)+{H}_{2}\frac{\partial u}{\partial r}+\frac{uv}{r+R}\right]={\mu }_{e}\left(\frac{{\partial }^{2}{H}_{1}}{\partial {r}^{2}}+\frac{1}{r+R}\frac{\partial {H}_{1}}{\partial r}-\frac{{H}_{1}}{{(r+R)}^{2}}\right),$$

6 $$\frac{R}{r+R}\left(u\frac{\partial T}{\partial s}\right)+v\frac{\partial T}{\partial r}=\frac{{k}_{f}}{{(p{c}_{p})}_{f}}\,\left(\frac{{\partial }^{2}T}{\partial {r}^{2}}+\frac{1}{r+R}\frac{\partial T}{\partial r}\right)-\frac{1}{{(p{c}_{p})}_{f}}\frac{1}{r+R}\frac{\partial }{\partial r}[(r+R){q}_{r}]+\frac{Q}{{(p{c}_{p})}_{f}}(T-{T}_{\infty }),$$

7 $$v\frac{\partial N}{\partial r}+\frac{Ru}{r+R}\frac{\partial N}{\partial s}=\frac{{\gamma }^{\ast }}{{\rho }_{f}\,j}\left(\frac{{\partial }^{2}N}{\partial {r}^{2}}+\frac{1}{r+R}\frac{\partial N}{\partial r}\right)-\frac{{k}^{\ast }}{{\rho }_{f}\,j}\left(\frac{\partial u}{\partial r}+2N+\frac{u}{r+R}\right),$$

Subjected to the boundary conditions

$$u=as+L\left(\frac{\partial u}{\partial r}-\frac{u}{r+R}\right),\,v=0,\,T={T}_{w}=\frac{As}{l},\,\frac{\partial {H}_{1}}{\partial r}={H}_{2}=0,\,at\,r=0,$$

8 $$u\to 0,\,\frac{\partial u}{\partial r}\to 0,\,T\to {T}_{\infty },\,{H}_{1}\to {H}_{e}(s)={H}_{0}s,\,as\,r\to \infty ,$$

Here $u,v,{H}_{1},{H}_{2}$ are the longitudinal and transverse components of velocity and magnetic field taken in s and r-direction respectively. ${\gamma }^{\ast }$ is spin gradient viscosity as is expressed as ${\gamma }^{\ast }=\left(\mu +\frac{{k}^{\ast }}{2}\right),\,j=\mu \left(1+\frac{{K}_{1}}{2}\right)j,\,j=\frac{v}{a}$, where ${K}_{1}=\frac{{k}^{\ast }}{\mu }$ is micropolar parameter. Also $p$ is pressure, $T$ is temperature, $l$ is slip length, $Q$ is volumetric heat rate depending upon temperature, ${k}^{\ast }$ is vortex viscosity and ${q}_{r}$ is radiative heat flux which can be expressed as

a $${q}_{r}=-\frac{4{\sigma }^{\ast }}{3{K}^{\ast }}\frac{\partial {T}^{4}}{\partial r},$$

Here ${\sigma }^{\ast }\,$is Stefan-Boltzmann constant and K* is mean proportion constant. Expanding ${T}^{4}$ about ${T}_{\infty }$ and neglecting higher powers i.e,

b $${T}^{4}\approx 4{T}_{\infty }^{3}T-3{T}_{\infty }^{3},$$

By applying above mentioned approximations (a) and (b) Eq. (6) turned into

9 $$\frac{R}{r+R}\left(u\frac{\partial T}{\partial s}\right)+v\frac{\partial T}{\partial r}=\frac{1}{Pr}\frac{{k}_{nf}}{{(p{c}_{p})}_{nf}}\left(1+Rd\frac{{k}_{f}}{{k}_{nf}}\right)\,\left(\frac{{\partial }^{2}T}{\partial {r}^{2}}+\frac{1}{r+R}\frac{\partial T}{\partial r}\right)+\frac{Q}{{(p{c}_{p})}_{nf}}(T-{T}_{\infty }),$$

In which $Pr={v}_{f}/{\alpha }_{f}$ and $Rd=16{\sigma }^{\ast }{T}_{\infty }^{3}/3{k}_{f}{K}^{\ast }$ are Prandtl number and Radiation parameter respectively. Following are the similarity transformation used in the analysis

10 $$\begin{array}{rcl}\eta  & = & \sqrt{\frac{a}{{v}_{f}}}r,\,u=asf{\prime} (\eta ),\,v=-\,\frac{R}{r+R}\sqrt{a{v}_{f}}\,f(\eta ),\,p={\rho }_{f}{a}^{2}{s}^{2}P(\eta ),\\  &  & {H}_{1}={H}_{0}sg{\prime} (\eta ),\,{H}_{2}=-\,{H}_{0}\frac{R}{r+R}\sqrt{\frac{{v}_{f}}{a}}g(\eta ),\,\theta (\eta )=\frac{T-{T}_{\infty }}{{T}_{w}-{T}_{\infty }},\\  &  & \chi (\eta )=\frac{C-{C}_{\infty }}{{C}_{w}-{C}_{\infty }}\,N=as\sqrt{\frac{a}{{v}_{f}}}q(\eta )\,and\,T={T}_{\infty }-\,\frac{As}{l}\,\theta (\eta )\end{array}$$

Using above transformations, Eqs. (1) and (2) are identically satisfied and Eqs. (3–7), (9), (10) simplify to

11 $$P{\prime} (\eta )={A}_{2}\frac{{(f{\prime} )}^{2}}{\eta +k},$$

12 $$\begin{array}{rcl}\frac{{\rho }_{f}}{{\rho }_{hnf}}\left(\frac{2k}{\eta +k}\right)P & = & \frac{{\rho }_{f}}{{\rho }_{hnf}}\left(\frac{1}{{(1-{\phi }_{1})}^{2.5}{(1-{\phi }_{2})}^{2.5}}+{K}_{1}\right)\left(f\prime\prime\prime +\frac{1}{\eta +k}f{\prime\prime} -\frac{1}{{(\eta +k)}^{2}}f{\prime} \right)\\  &  & -\frac{k}{\eta +k}\left({f{\prime} }^{2}-ff{\prime\prime} -\frac{ff{\prime} }{\eta +k}\right)+\frac{\beta k}{\eta +k}\left({g}^{2}-gg{\prime\prime} -\frac{gg{\prime} }{\eta +k}\right)-{K}_{1}\frac{{\rho }_{f}}{{\rho }_{hnf}}q(\eta ),\end{array}$$

13 $$\frac{k}{\eta +k}(f{\prime\prime} g-fg{\prime\prime} )-\frac{k}{{(\eta +k)}^{2}}\left(\gamma gg{\prime} -\frac{1}{\gamma }ff{\prime} \right)=\lambda \,\left(g\prime\prime\prime +\frac{g{\prime\prime} }{\eta +k}-\frac{g{\prime} }{{(\eta +k)}^{2}}\right),$$

14 $$\frac{{A}_{3}}{Pr{A}_{4}}\left(1+\frac{Rd}{{A}_{3}}\right)\left(\theta {\prime\prime} +\frac{\theta {\prime} }{\eta +k}\right)+\frac{D}{{A}_{4}}\theta =\frac{k}{\eta +k}(f{\prime} \theta -f\theta {\prime} ),$$

15 $$\frac{k}{\eta +k}(f{\prime} q-fq{\prime} )=\frac{{\rho }_{f}}{{\rho }_{hnf}}\left(\frac{1}{{(1-{\phi }_{1})}^{2.5}{(1-{\phi }_{2})}^{2.5}}+\frac{{K}_{1}}{2}\right)\left(q{\prime\prime} +\frac{q{\prime} }{\eta +k}\right)-\frac{{\rho }_{f}}{{\rho }_{hnf}}K\left(f{\prime\prime} +2N+\frac{f{\prime} }{\eta +k}\right),$$

Boundary conditions are transformed as

16 $$\begin{array}{l}f(0)=0,\,f{\prime} (0)=1+K\left(f{\prime\prime} (0)-\frac{1}{k}f{\prime} (0)\right),\,g(0)=0,\,g{\prime\prime} (0)=0,\,\theta (0)=1,\,q(0)=-nf{\prime\prime} (0),\\ f{\prime} (\eta )\to 0,\,f{\prime\prime} (\eta )\to 0,\,g{\prime} (\eta )\to 1,\,\theta (\eta )\to 0,\,q(\eta )\to 0,\,as\,\eta \to \infty .\end{array}$$

Here $\beta ,Pr,k,D,\lambda ,K\,and\,\gamma$ are the dimensionless magnetic parameter, Prandtl number, curvature parameter, heat generation parameter, reciprocal magnetic Prandtl number, slip parameter and dimensionless parameter respectively and are defined as

17 $$k=R\sqrt{\frac{a}{{v}_{f}}},\,\beta =\frac{{\mu }_{e}}{4\pi {\rho }_{f}}{\left(\frac{{H}_{0}}{a}\right)}^{2},\,Pr=\frac{{v}_{f}{(p{c}_{p})}_{f}}{{k}_{f}},\,K=L\sqrt{\frac{a}{{v}_{f}}},\,\lambda =\frac{{\mu }_{e}}{{\mu }_{f}},\,\gamma =\frac{{H}_{0}}{a}$$

Differentiating Eq. (12) and using (11) to eliminate pressure, we get the following equation

18 $$\begin{array}{c}{f}^{iv}+\frac{2f\prime\prime\prime }{\eta +k}-\frac{f{\prime\prime} }{{(\eta +k)}^{2}}+\frac{f{\prime} }{{(\eta +k)}^{3}}+\frac{{\rho }_{f}}{{\rho }_{hnf}}\left(\frac{1}{{(1-{\phi }_{1})}^{2.5}{(1-{\phi }_{2})}^{2.5}}+\frac{{K}_{1}}{2}\right)\left[\left(\frac{k}{\eta +k}\right)(ff\prime\prime\prime -f{\prime} f{\prime\prime} )\right.\\ +\left(\frac{k}{{(\eta +k)}^{2}}\right)(ff{\prime\prime} -{f{\prime} }^{2})-\left(\frac{k}{{(\eta +k)}^{3}}\right)ff{\prime} +\frac{\beta k}{(\eta +k)}\left(g{\prime} g{\prime\prime} -gg\prime\prime\prime +\frac{gg{\prime} }{{(\eta +k)}^{2}}\right.\\ \left.\left.-\frac{1}{(\eta +k)}({g{\prime} }^{2}+gg{\prime\prime} )\right)\right]-\left(\frac{1}{{(1-{\phi }_{1})}^{2.5}{(1-{\phi }_{2})}^{2.5}}+\frac{{K}_{1}}{2}\right){K}_{1}\left(q{\prime\prime} +\frac{q{\prime} }{(\eta +k)}\right)=0,\end{array}$$

The pressure profile can be computed from (12) as

19 $$P(\eta )=\frac{(\eta +k)}{2k}\,\frac{{\rho }_{hnf}}{{\rho }_{f}}\,[\frac{{\rho }_{f}}{{\rho }_{hnf}}(\frac{1}{{(1-{\phi }_{1})}^{2.5}{(1-{\phi }_{2})}^{2.5}}+\frac{{K}_{1}}{2})(f\prime\prime\prime +\frac{1}{\eta +k}f{\prime\prime} -\frac{1}{{(\eta +k)}^{2}}f{\prime} )-\frac{k}{\eta +k}({f{\prime} }^{2}-ff{\prime\prime} -\frac{ff{\prime} }{\eta +k})+\frac{\beta k}{\eta +k}({g{\prime} }^{2}-gg{\prime\prime} -\frac{gg{\prime} }{\eta +k})-{K}_{1}\frac{{\rho }_{f}}{{\rho }_{hnf}}q{\prime} (\eta )]$$

Substantial measurable quantities Shear stress $C{f}_{s}$ and heat flux rate $N{u}_{s}$ are defined as

20 $${C}_{f}=\frac{{\tau }_{rs}}{{\rho }_{hnf}\,{u}_{w}^{2}},\,{N}_{us}=\frac{s{q}_{w}}{{k}_{f}({T}_{w}-{T}_{\infty })},$$

where the heat transfer ${q}_{w}$ and wall friction ${\tau }_{rs}$ along $s\,-$ direction is expressed as

21 $${\tau }_{rs}=({\mu }_{hnf}+{k}^{\ast }){\left(\frac{\partial u}{\partial r}+\frac{u}{r+R}+{k}^{\ast }N\right)}_{r=0},\,{q}_{w}=-\,{k}_{hnf}{\left(\frac{\partial T}{\partial r}\right)}_{r=0}$$

The Skin friction coefficient and Nusselt number is evaluated as

22 $$R{e}^{\frac{1}{2}}\,C{f}_{s}=\frac{{{\rm{\rho }}}_{{\rm{f}}}}{{{\rm{\rho }}}_{{\rm{hnf}}}}\left(\frac{1}{{(1-{\Phi }_{2})}^{5/2}{(1-{\Phi }_{1})}^{5/2}}+{{\rm{K}}}_{1}\right)\left(f{\prime\prime} (0)+\frac{f{\prime} (0)}{\eta +k}-{K}_{1}nf{\prime\prime} (0)\right),$$

23 $$R{e}^{-1/2}N{u}_{s}=-\frac{{k}_{hnf}}{{k}_{f}}\left(1+Rd\frac{{k}_{f}}{{k}_{hnf}}\right)\theta {\prime} (0),$$

where $Re=a{s}^{2}/{v}_{f}$ is local Reynold number.

## Results and discussion

The magnetized micropolar base fluid with nanoparticles over a curved surface is considered. The system of differential equations is solved through numerical technique. In this segment of problem, the impact of different parameters is examined by means of graphs plotted for numerically calculated velocity, temperature and micro rotation for distinct values of curvature parameter $k$, velocity slip parameter $K$, magnetic parameter $\beta$, reciprocal magnetic Prandtl number $\lambda$, CNTs volumetric fraction for hybrid fluid ${\phi }_{2}$ and dimensionless parameter $\gamma$. The range of the physical parameters are taken from the literature (see Refs. ^44–46^). Such numerical values are used in the experimental data. Our results are shown to be the most reliable and has good agreement with the decay literature as shown in Table 1.

**Table 1:** Validity of our results with decay when the rest of the physical parameters are zero.

S	Nadeem *et al*.^46^	Wang^47^	Present study
0.0	1.231588	1.232588	1.23159
0.1	1.143556	1.14656	1.14349
0.2	1.050613	1.05113	1.05039
0.5	0.712510	0.71330	0.71249
1	0	0	0
2	−1.868321	−1.88731	−1.86822
5	−10.25234	−10.26475	−10.2518

### Numerical analysis

Table 2 delineates the strong concentration of Nusslet number and skin friction coefficients for the base fluids water and propylene glycol. The variation of physical parameters use in this study, i.e. $k,\,\lambda ,\,Rd,\,\gamma ,\,\beta ,\,D,\,K,\,{\Phi }_{2}$ and ${K}_{1}$ are shown below in Table 2. It is revealed that enhancing curvature parameter results which are declining $R{e}^{1/2}\,C{f}_{s}$ for both water and propylene glycol. The magnitude of the skin fraction $-R{e}^{-1/2}\,N{u}_{s}$ reduced for enhancing the values of curvature parameter $k$. The impact of a reciprocal magnetic Prandtl number $\lambda$ is worth noting, increase in $\lambda$ leads to increase in $R{e}^{1/2}\,C{f}_{s}$ for both base fluids and opposite behavior is seen for $-R{e}^{-1/2}\,N{u}_{s}$. The neutral behavior of skin friction coefficient is examined when $Rd$ is increased, i.e.t remained constant for both water and propylene glycol, but Nusselt number indicated a decreasing behavior for both base fluids. It is demonstrated that $R{e}^{1/2}\,C{f}_{s}$ decreases for water and increases for propylene glycol as the value of $\gamma$ upsurges where as Nusselt number increases for propylene glycol and declines for water when $\gamma$ is enhanced. Table 2 disclosed that the escalated value of $\beta$ cause $R{e}^{1/2}\,C{f}_{s}$ to elevate for both base fluids while $-R{e}^{-1/2}\,N{u}_{s}$ presents no variation. The impact of enhancing $D$ is that skin friction coefficient larger for propylene glycol and also for water whereas. $-R{e}^{-1/2}\,N{u}_{s}$ depicts an increase for water and a decrement for propylene glycol. The slip parameter $K$ has a vital impact on skin friction and Nusselt number. When $K$ is escalated then $R{e}^{1/2}\,C{f}_{s}$ decreases for both base fluids and $-R{e}^{-1/2}\,N{u}_{s}$ results in drastic increament. Increase in value of ${\Phi }_{2}$ display a sharp reduction in $R{e}^{1/2}\,C{f}_{s}$ for water and propylene glycol while $-R{e}^{-1/2}\,N{u}_{s}$ upswings for both base fluids. Lastly, it is reported that skin friction coefficient upturns for water and propylene glycol as micropolar parameter ${K}_{1}$ increases. On the other side Nusselt number declines for water and grows for propylene glycol.

**Table 2:** Numerical outcomes of ${R}{{e}}^{1/2}\,C{f}_{s}$ and $-{\mathrm{Re}}^{-1/2}\,N{u}_{s}$ when $n=0.5$.

$k$	$\lambda$	$Rd$	$\gamma$	$\beta$	$D$	$K$	${\Phi }_{2}$	${K}_{1}$	Water		Propylene glycol	
$k$	$\lambda$	$Rd$	$\gamma$	$\beta$	$D$	$K$	${\Phi }_{2}$	${K}_{1}$	${\mathrm{Re}}^{1/2}\,C{f}_{s}$	${\mathrm{Re}}^{-1/2}\,N{u}_{s}$	${\mathrm{Re}}^{1/2}\,C{f}_{s}$	${\mathrm{Re}}^{-1/2}\,N{u}_{s}$
0.4	0.5	0.5	0.4	0.5	0.5	0.4	0.05	0.8	3.2667	−1.1789	3.2256	−1.1879
0.6	—	—	—	—	—	—	—	—	2.8465	−1.1579	2.8014	−1.1769
0.8	—	—	—	—	—	—	—	—	2.1095	−1.1479	2.0809	−1.1579
0.8	0.1	—	—	—	—	—	—	—	1.2082	−0.6538	0.9514	−0.5714
—	0.3	—	—	—	—	—	—	—	1.3324	−0.7640	1.3144	−0.7640
—	0.5	—	—	—	—	—	—	—	2.1095	−1.1486	2.0809	−1.1486
—	0.5	0.2	—	—	—	—	—	—	2.1095	−0.8484	2.0809	−0.8484
—	—	0.3	—	—	—	—	—	—	2.1095	−0.9484	2.0809	−0.9485
—	—	0.4	—	—	—	—	—	—	2.1095	−1.0485	2.0809	−1.0485
—	—	0.5	—	—	—	—	—	—	2.1095	−1.1486	2.0809	−1.1486
—	—	0.5	0.1	—	—	—	—	—	2.2223	−1.1479	2.0867	−1.1486
—	—	—	0.2	—	—	—	—	—	2.1190	−1.1479	2.0906	−1.1479
—	—	—	0.3	—	—	—	—	—	2.1099	−1.1479	2.0938	−1.1479
—	—	—	0.4	0.1	—	—	—	—	2.0325	−1.1486	2.0039	−1.1486
—	—	—	—	0.3	—	—	—	—	2.0793	−1.1486	2.0514	−1.1486
—	—	—	—	0.5	—	—	—	—	2.1095	−1.1486	2.0809	−1.1486
—	—	—	—	0.5	0.1	—	—	—	2.1095	−1.1486	1.8078	−0.9596
—	—	—	—	—	0.2	—	—	—	2.1095	−1.1486	2.0809	−1.1486
—	—	—	—	—	0.3	—	—	—	2.0782	−1.1489	2.0809	−1.1486
—	—	—	—	—	0.4	—	—	—	2.0681	−1.1479	2.0809	−1.1486
—	—	—	—	—	0.5	0.1	—	—	2.2828	−1.1489	2.5369	−1.1489
—	—	—	—	—	—	0.3	—	—	2.2459	−1.1485	2.3203	−1.1483
—	—	—	—	—	—	0.5	—	—	2.0470	−1.1486	2.0194	−1.1482
—	—	—	—	—	—	0.4	0.01		2.1933	−1.2288	2.1682	−1.2288
—	—	—	—	—	—	—	0.03		2.1498	−1.1872	2.1229	−1.1872
—	—	—	—	—	—	—	0.05		2.1095	−1.1486	2.0809	−1.1486
—	—	—	—	—	—	—	0.05	0.4	1.6976	−1.1483	1.4668	−1.1489
—	—	—	—	—	—	—	—	0.6	1.9218	−1.1485	2.0157	−1.1483
—	—	—	—	—	—	—	—	0.8	2.1095	−1.1486	2.0809	−1.1481

### Velocity distribution

**Figure 2:**
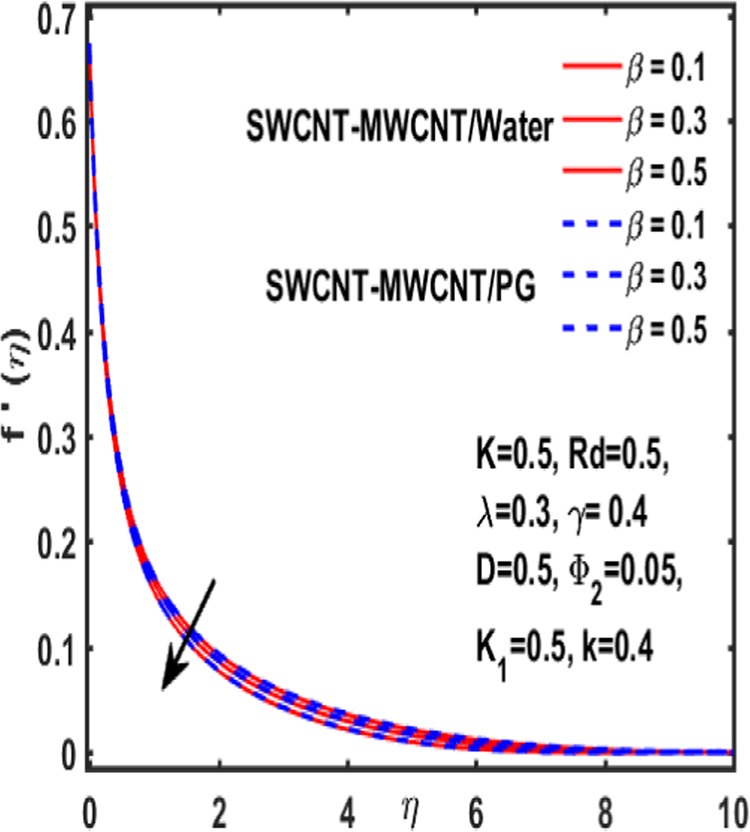
Variation of ${\rm{\beta }}$ on velocity.

**Figure 3:**
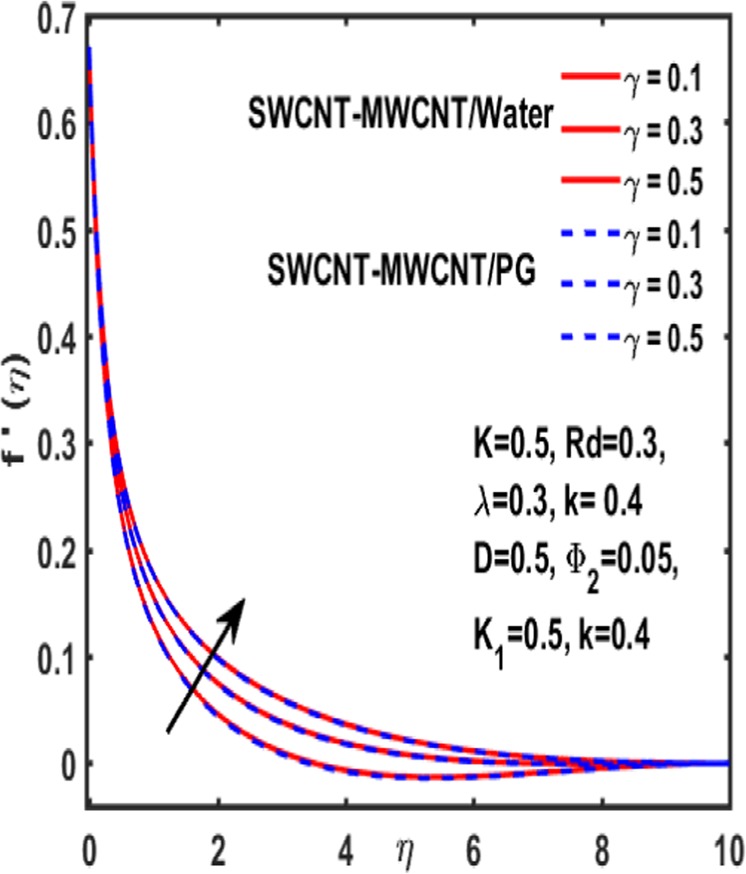
Variation of ${\rm{\gamma }}$ on velocity.

**Figure 4:**
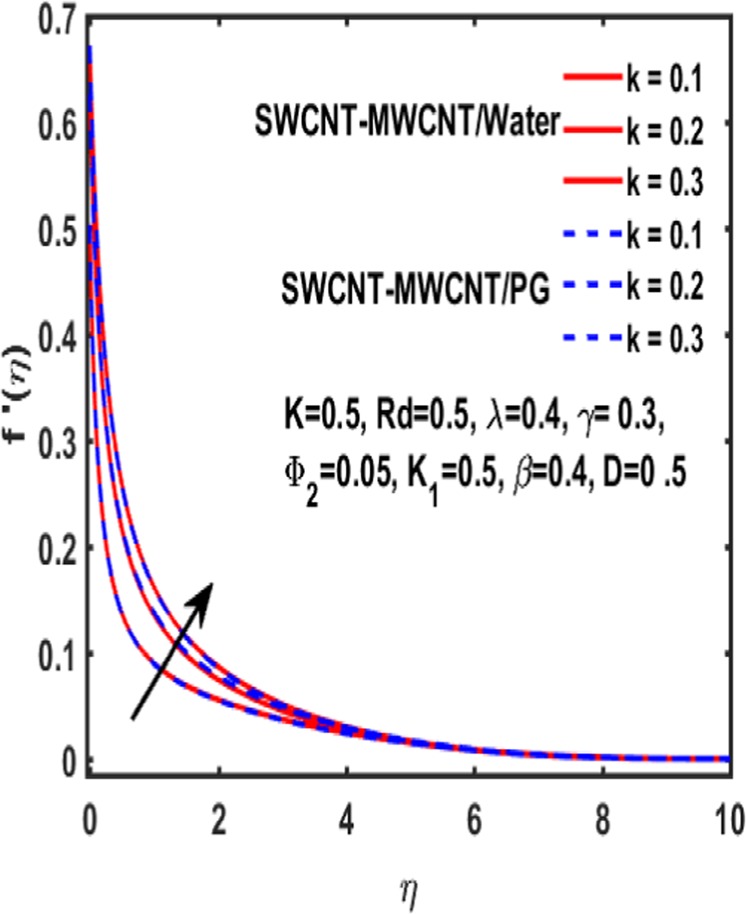
Variation of k on velocity.

**Figure 5:**
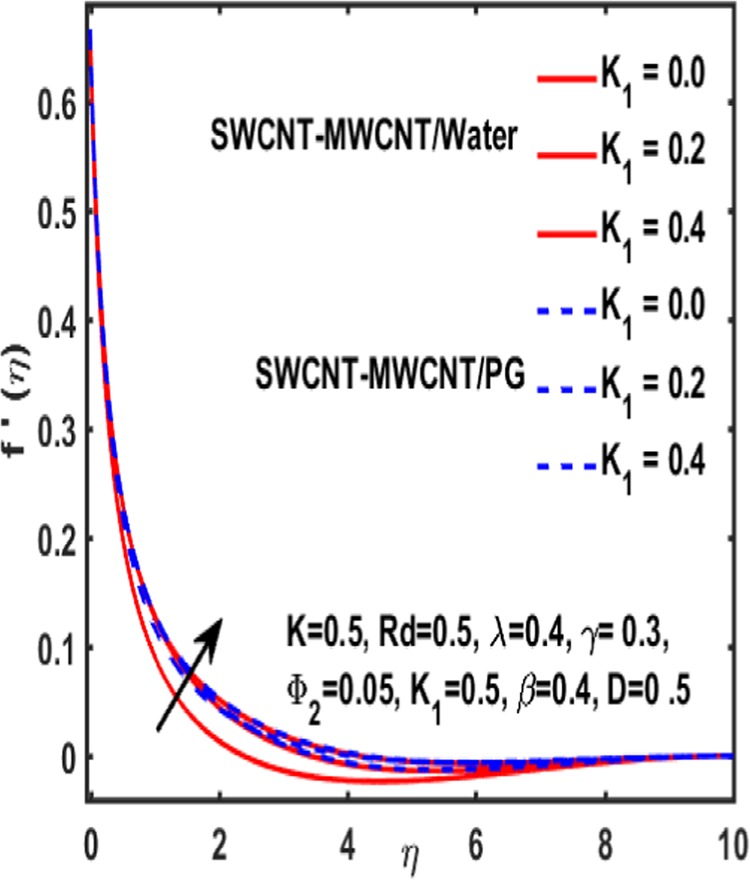
Variation of ${{\rm{K}}}_{1}$ on velocity.

**Figure 6:**
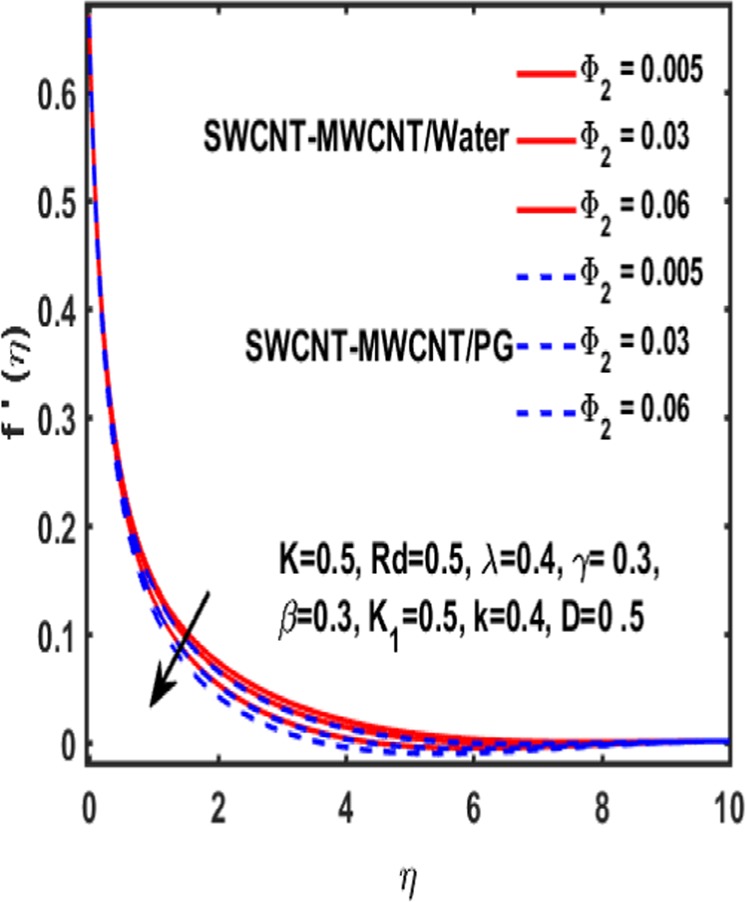
Impact of ${\Phi }_{2}$ on velocity.

The effect of curvature parameter $k$, velocity slip parameter $K$, magnetic parameter $\beta$, reciprocal magnetic Prandtl number $\lambda$, solid nanoparticle ${{\boldsymbol{\Phi }}}_{2}$, dimensionless parameter $\gamma$ and material parameter ${K}_{1}$ are studied through graphs drawn in Figs. 2–6. Figure 2 reveals the consequence of increasing $\beta$ on velocity profile. It is observed that an increment in magnetic parameter $\beta$ declines the velocity function for both types of base fluids i.e. water and Propylene glycol. The effect of dimensionless parameter $\gamma$ on fluid flow is examined through Fig. 3. From this figure it is noted that there is a decrement in velocity profile whenever $\gamma$ is increased. Figure 4 is drawn to depict the behavior of velocity when an uplift is done in curvature parameter $k$. This graph indicates that velocity profile accelerates with an increase in curvature parameter. Figure 5 is plotted to explain the impact of material parameter ${K}_{1}$ on velocity profile of the hybrid nano fluid flow. A downfall in velocity function is observed when material parameter is increased. It is witnessed that presence of induced magnetic field plays a vital role in accelerating fluid flow. Figure 6 is plotted to demonstrate the effect of nanoparticle volume fraction ${{\boldsymbol{\Phi }}}_{2}$ on velocity.

**Nomanclature**

**Table:** 

$k$	Curvature parameter	${{\rm{\kappa }}}_{{\rm{f}}}$	Thermal conductivity of fluid
$K$	Velocity slip	${\rm{{\rm K}}}{\rm{nf}}$	Thermal conductivity of nanofluid
${K}_{1}$	Micropolar parameter	${\rm{{\rm K}}}{\rm{hnf}}$	Thermal conductivity of hybrid nanofluid
$Rd$	Radiation parameter	${\rm{{\rm M}}}{\rm{hnf}}$	Viscosity of hybrid nanofluid
$\gamma$	Dimensionless parameter	${\rm{{\rm M}}}{\rm{nf}}$	Viscosity of nanofluid
${\Phi }_{2}$	Solid nanoparticle	$({\rm{\rho }}{\rm{Cp}}){\rm{nf}}$	Heat capacity of nanofluid
$\lambda$	Reciprocal Magnetic Prandtl number	${\rm{{\rm A}}}{\rm{hnf}}$	thermal diffusivity of hybrid nanofluid
$\beta$	Magnetic parameter	${\rm{{\rm A}}}{\rm{nf}}$	thermal diffusivity of nanofluid
$D$	Heat source parameter	${\rm{\nu }}{\rm{hnf}}$	Hybrid nanofluid kinematic Viscosity
$n$	Microgyration parameter	$({\rm{\rho }}{\rm{Cp}}){\rm{hnf}}$	Heat capacity of hybrid nanofluid

### Temperature distribution

**Figure 7:**
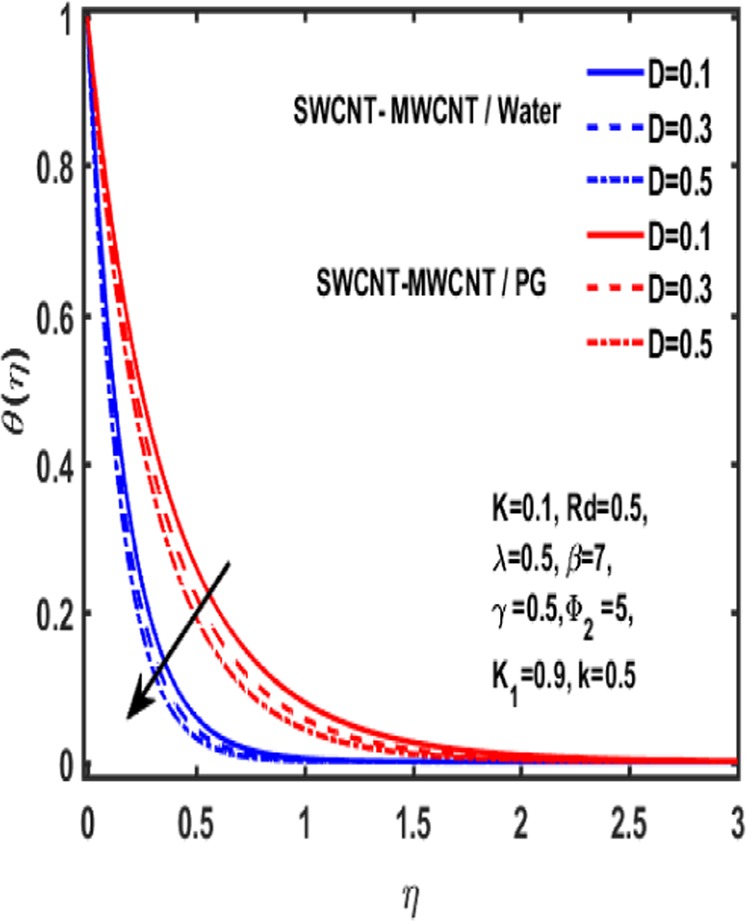
Variation of D on temperature.

**Figure 8:**
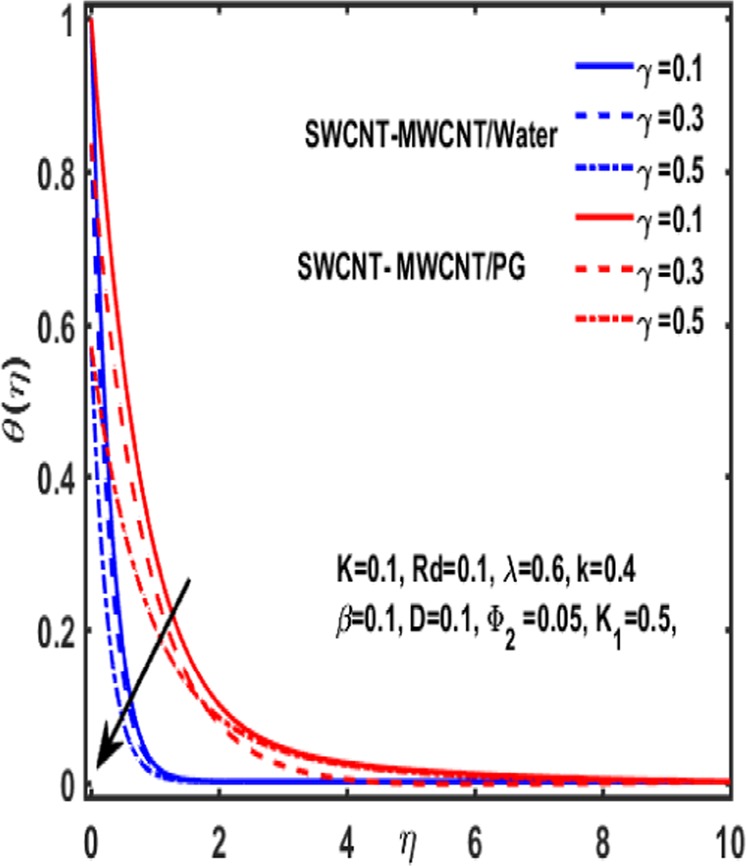
Influence of ${\rm{\gamma }}$ on temperature.

**Figure 9:**
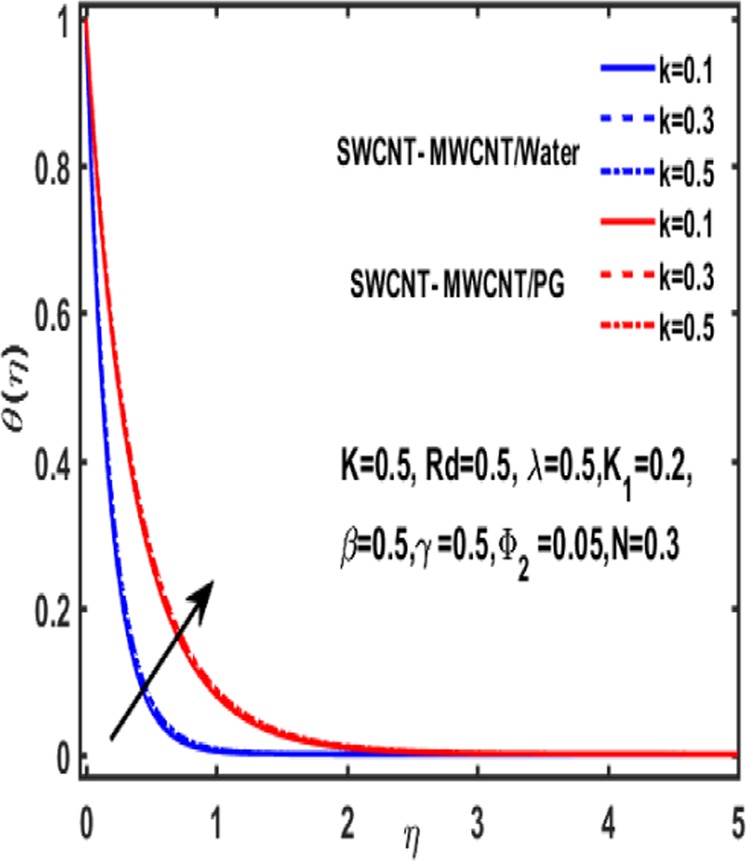
Variation of k on temperature.

**Figure 10:**
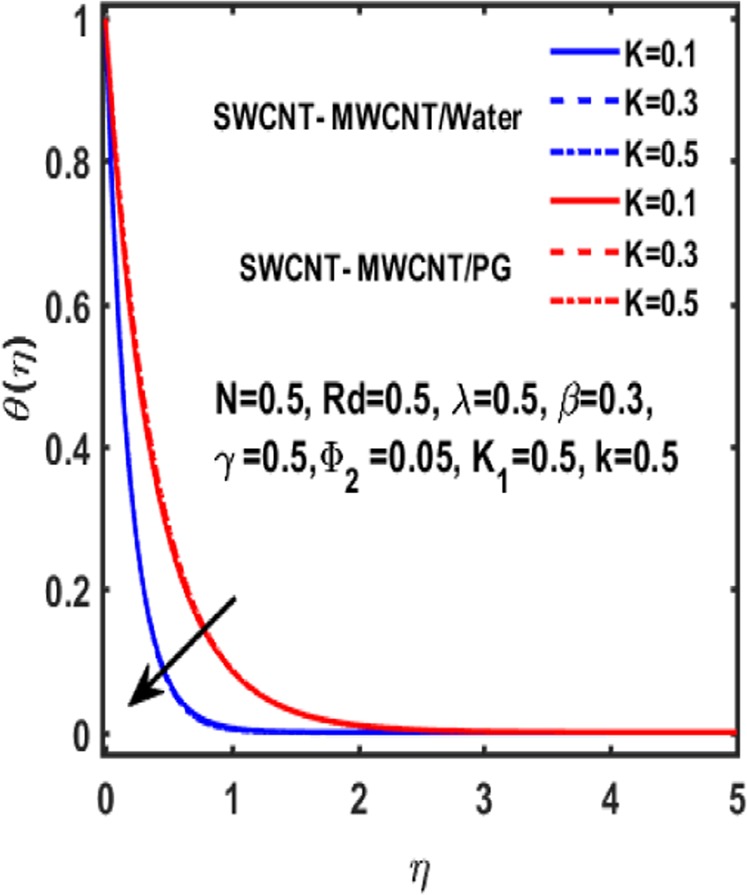
Impact of K on temperature.

**Figure 11:**
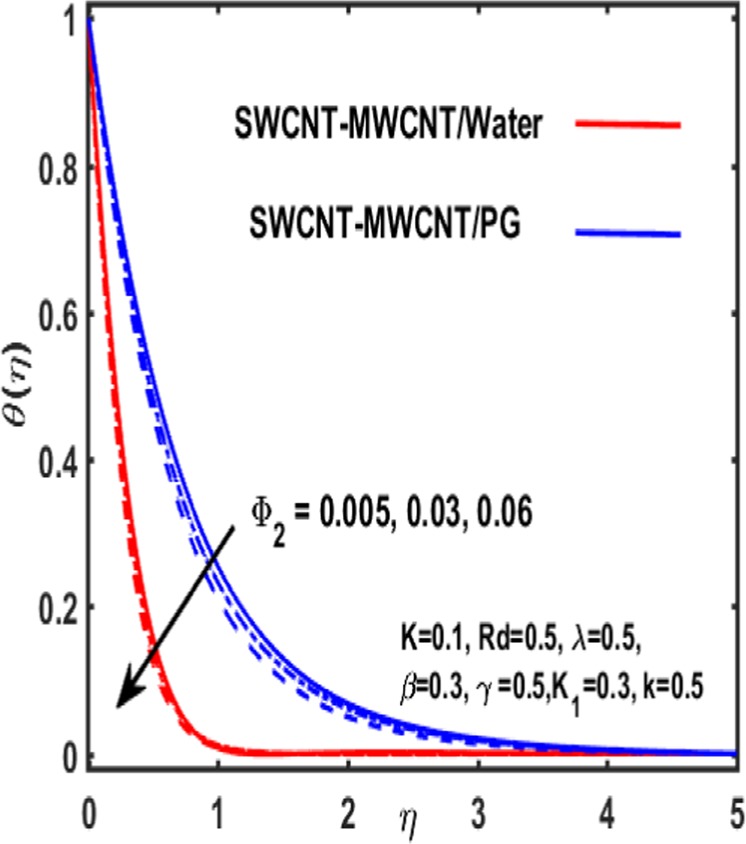
Impact of ${{\boldsymbol{\Phi }}}_{2}$ on ${\boldsymbol{\theta }}$.

**Figure 12:**
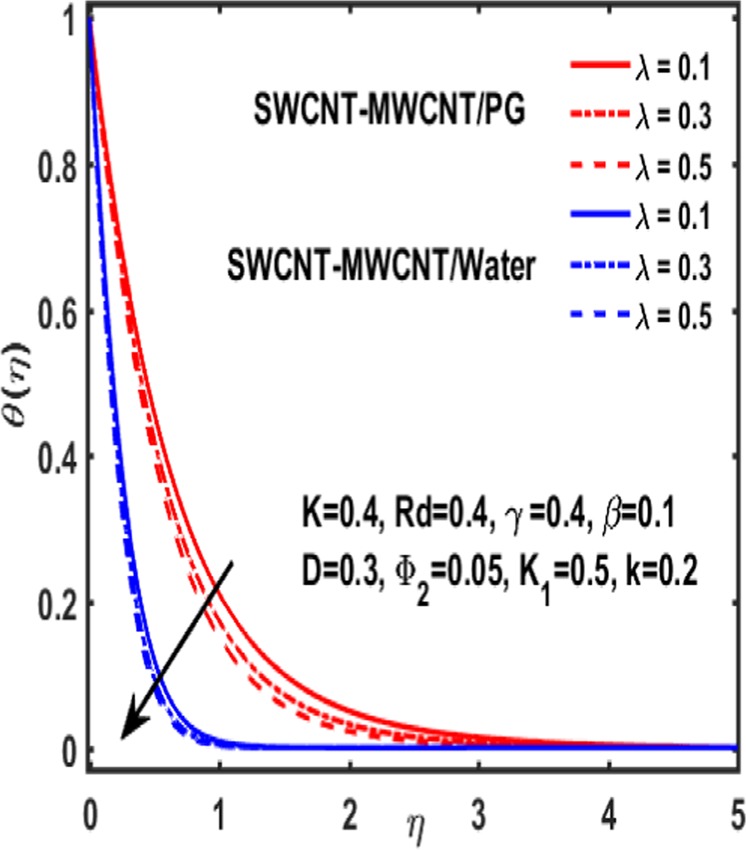
Influence of ${\rm{\lambda }}$ on ${\rm{\theta }}$.

Figures 7–12 are drawn for the ample interpretation of fluid flow temperature when CNTs are dispersed in micropolar hybrid nanofluid. In these figures blue lines depict the results for water and red lines represents propylene glycol respectively. The effect of heat generation parameter D on the temperature function which is displayed in Fig. 7. It is witnessed that decelerating behavior is shown by fluid temperature as the heat generation parameter up surges. Figure 8 is plotted to represent the impact of dimensionless parameter $\gamma$ on temperature gradient of fluid flow. It is observed that temperature profile is decreasing function of $\gamma$. Figure 9 demonstrates the effect of curvature parameter $k$ on temperature. It is revealed that temperature is enhanced with an increasing value of curvature parameter. The role of slip parameter $K$ is studied through Fig. 10 which demonstrates that enhancing the slip parameter $K$ results in drastic increase in temperature profile in case of propylene glycol and a decrease is reported in temperature when water is considered as base fluid. Figure 11 reveals the influence of ${\Phi }_{2}$ on the temperature gradient. It is seen that temperature function declines due to higher values of solind particles because the thermal boundary layer thikness redued. The impacts of ${\rm{\lambda }}$ on the temperature gradienct which see in Fig. 12. The temperature gradient declines due to higher values of ${\rm{\lambda }}$.

### Micropolar rotation distribution

**Figure 13:**
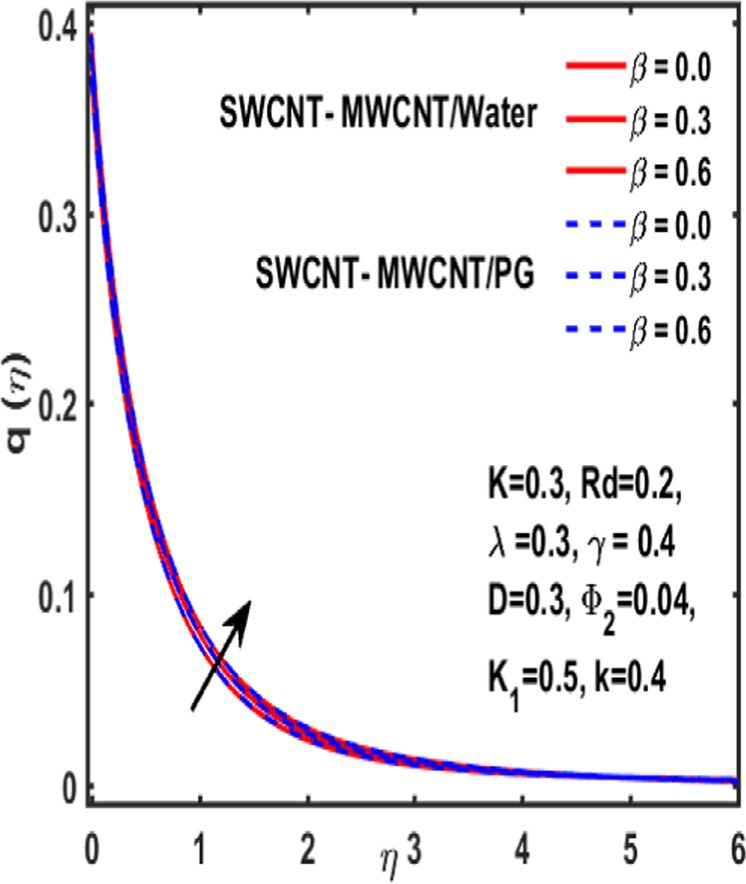
Variation of β on $q(\eta )$.

**Figure 14:**
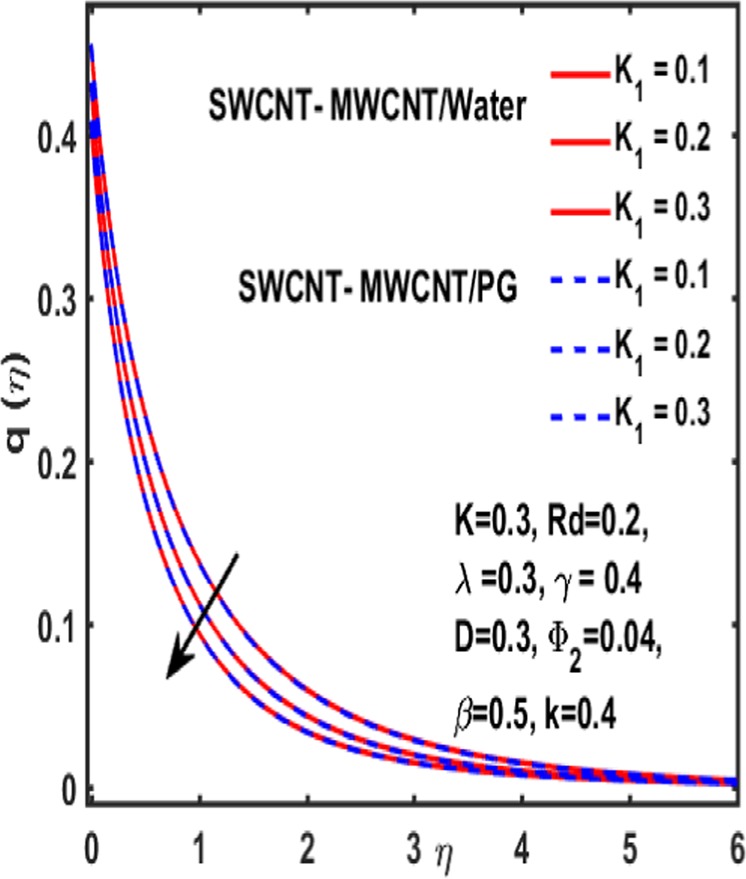
Impact of ${{\rm{K}}}_{1}$ on ${\boldsymbol{q}}({\boldsymbol{\eta }})$.

**Figure 15:**
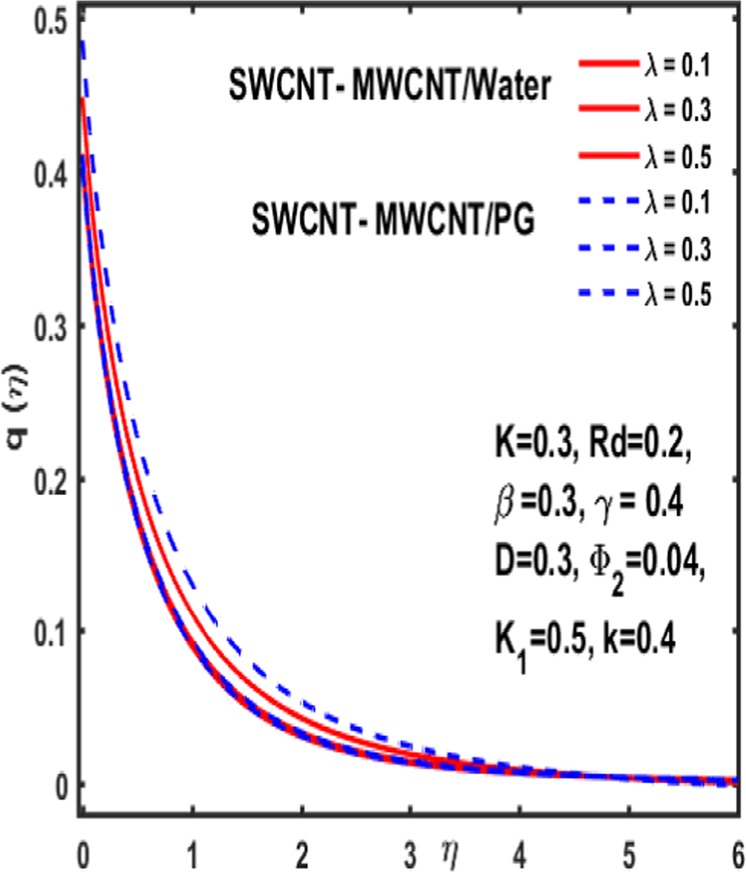
Influence of $\lambda$ on ${\boldsymbol{q}}({\boldsymbol{\eta }})$.

**Figure 16:**
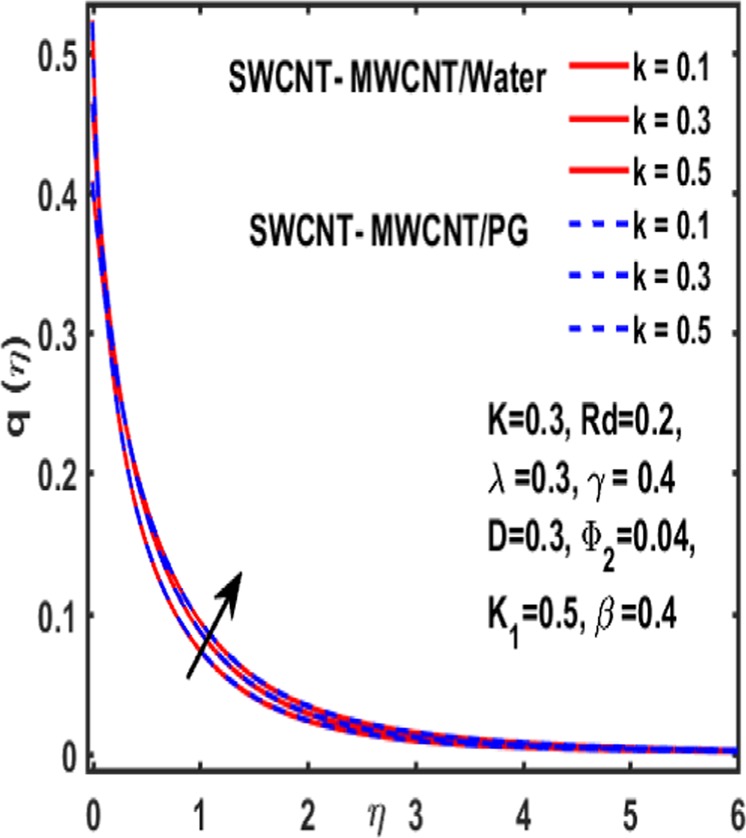
Impact of k on $q(\eta )$.

**Figure 17:**
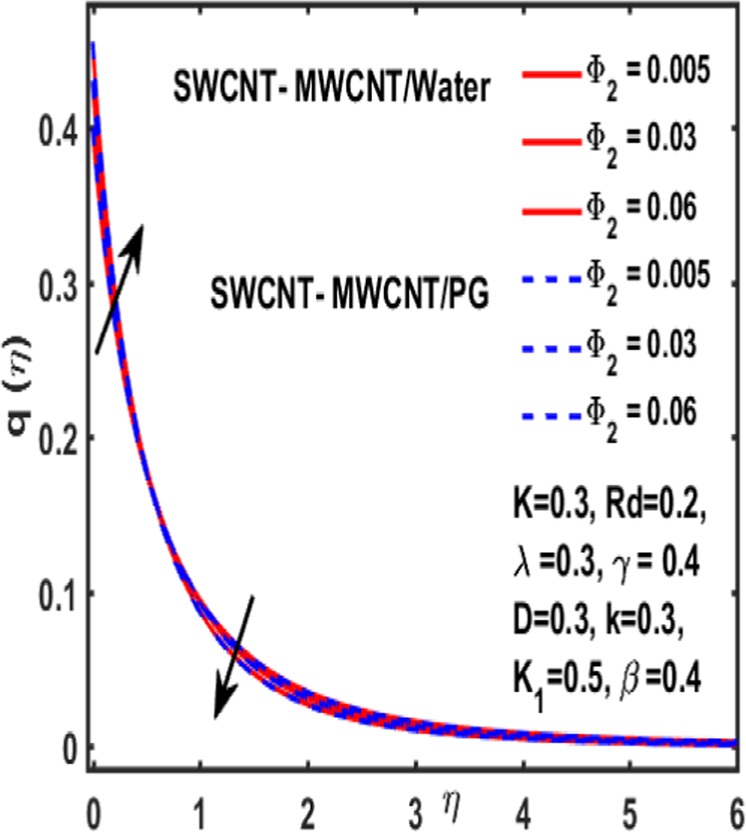
Variation of ${{\boldsymbol{\Phi }}}_{2}$ on ${\boldsymbol{q}}({\boldsymbol{\eta }})$.

**Figure 18:**
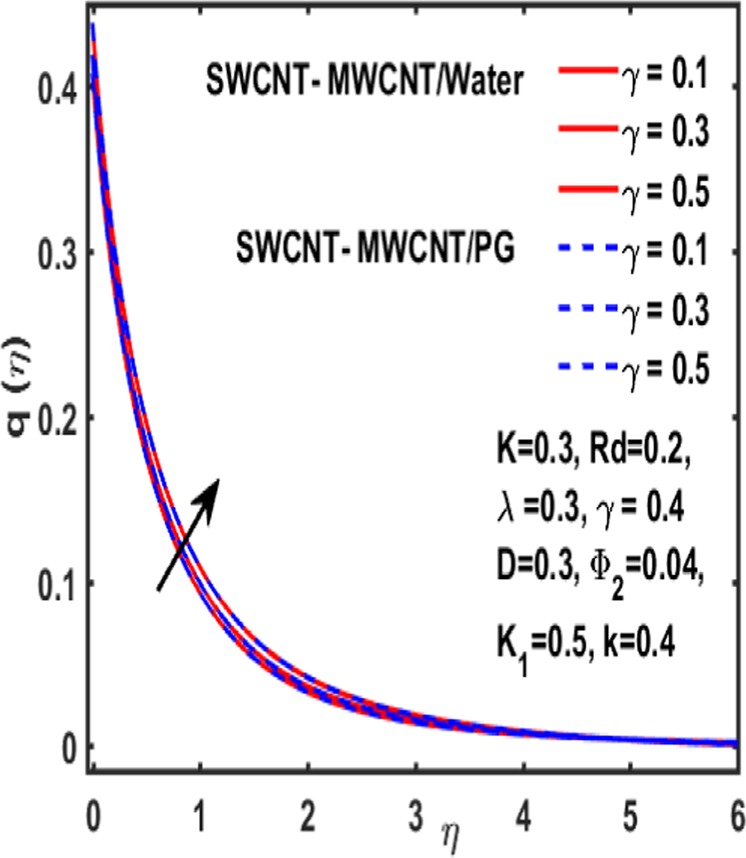
Influence of ${\boldsymbol{\gamma }}\,$on ${\boldsymbol{q}}({\boldsymbol{\eta }})$.

The influence of significant parameters $\beta ,\gamma ,{K}_{1},k\,,{\Phi }_{2}\,and\,\lambda$ on micropolar rotation ${\boldsymbol{q}}({\boldsymbol{\eta }})$ is analyzed through Fig. 13–18. Figure 13 is plotted to represent the influence of magnetic parameter $\beta$ on ${\boldsymbol{q}}({\boldsymbol{\eta }})$ and reveals that micropolar rotation is a declining function of magnetic parameter. The effect of material parameter ${K}_{1}$ is studied through Fig. 14. It shows that micropolar rotation decreases for an increasing value of ${K}_{1}$. Figure 15 illustrates the influence of reciprocal magnetic Prandtl number on ${\boldsymbol{q}}({\boldsymbol{\eta }})$. It is witnessed that for water the micropolar rotation is enhanced as material parameter increases while a decrement is observed in case of propylene glycol as the value of ${K}_{1}$ up surges. The impact of curvature parameter $k$ on ${\boldsymbol{q}}({\boldsymbol{\eta }})$ is examined through Fig. 16. It explains that curvature parameter enhances the curviness of micropolar rotation ${\boldsymbol{q}}({\boldsymbol{\eta }})$. Figure 17 is plotted to show the effect of volumetric fraction of nanoparticle ${\Phi }_{2}$ on ${\boldsymbol{q}}({\boldsymbol{\eta }})$. It is observed that micropolar rotation increases as volumetric fraction enhances. Finally, the impact of $\lambda$ on micropolar rotation ${\boldsymbol{q}}({\boldsymbol{\eta }})$ is demonstrated in Fig. 18. It is reported that enhancing the value of reciprocal magnetic Prandtl number results in shrinkage of curviness of ${\boldsymbol{q}}({\boldsymbol{\eta }})$.

### Effect on induced magnetic field

**Figure 19:**
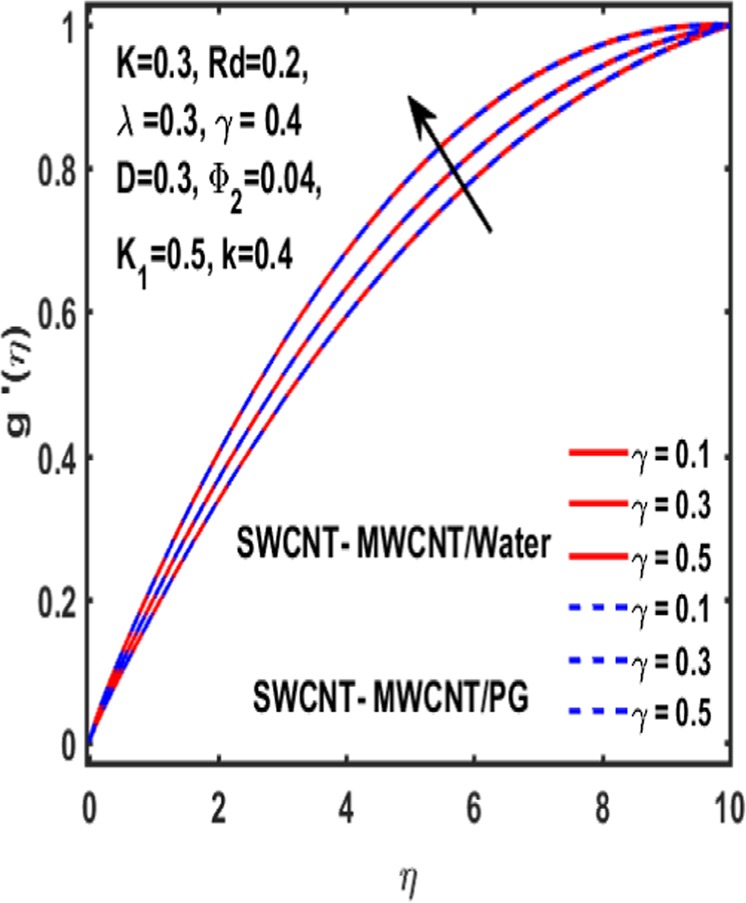
Influence ${\boldsymbol{\gamma }}$ of on ${\boldsymbol{g}}{\boldsymbol{{\prime} }}({\boldsymbol{\eta }})$.

**Figure 20:**
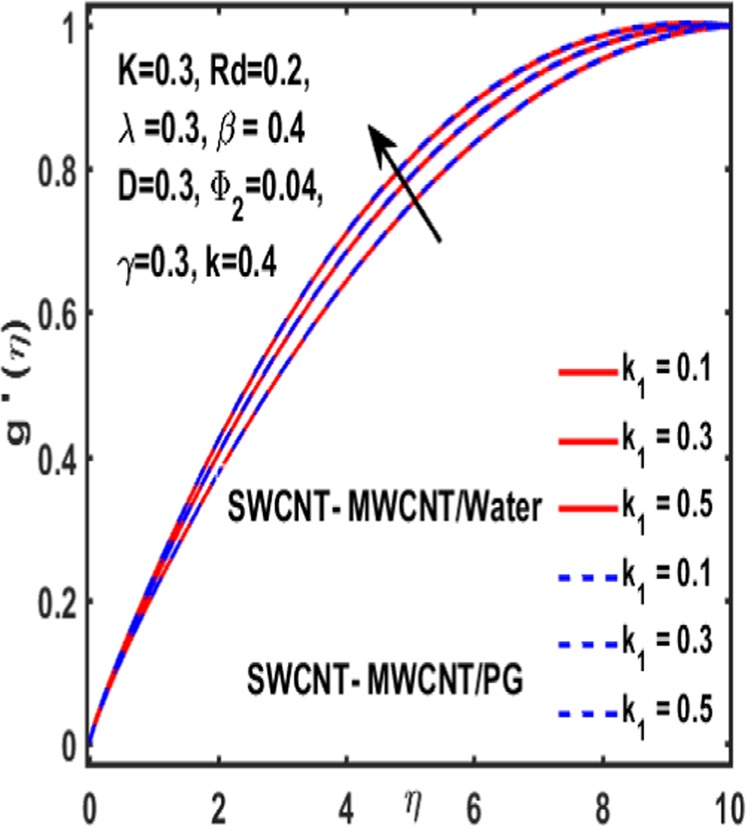
Influence ${{\boldsymbol{K}}}_{1}$ of on ${\boldsymbol{g}}{\boldsymbol{{\prime} }}({\boldsymbol{\eta }})$.

**Figure 21:**
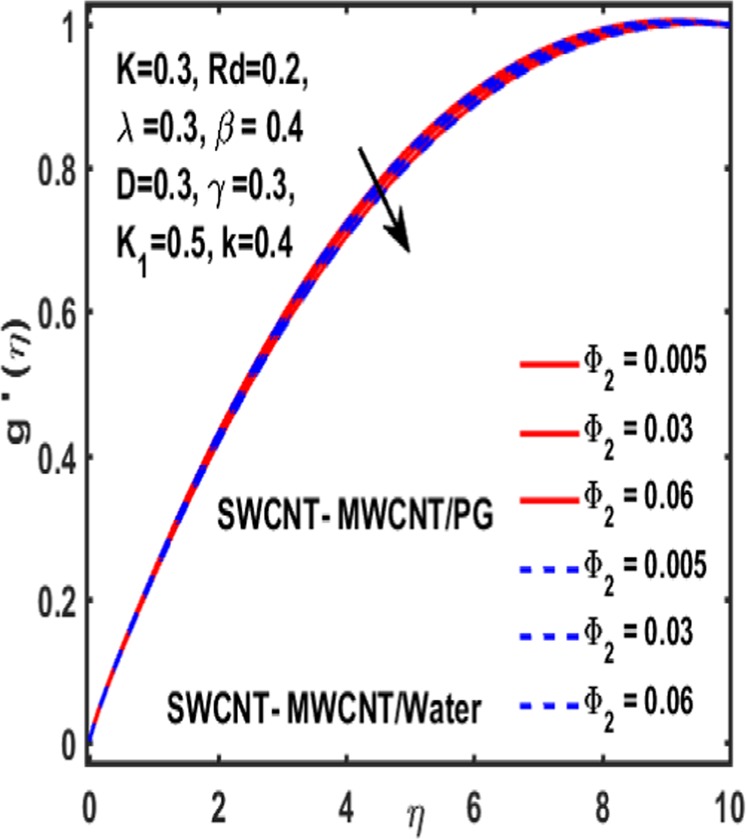
Influence ${{\boldsymbol{\Phi }}}_{2}$ of on ${\boldsymbol{g}}{\boldsymbol{{\prime} }}({\boldsymbol{\eta }})$.

**Figure 22:**
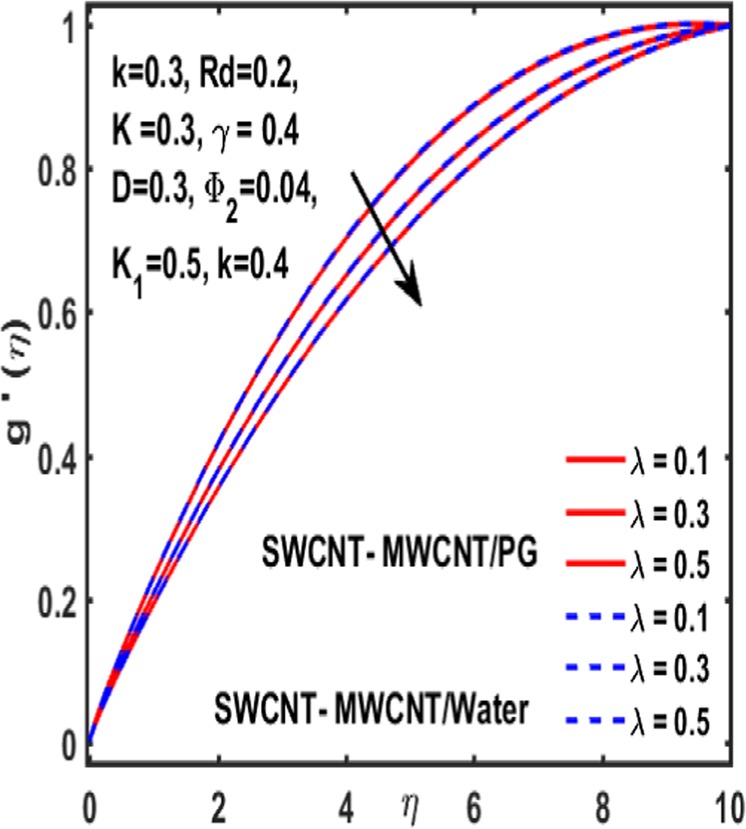
Influence ${\boldsymbol{\lambda }}$ of on ${\boldsymbol{g}}{\boldsymbol{{\prime} }}({\boldsymbol{\eta }})$.

**Figure 23:**
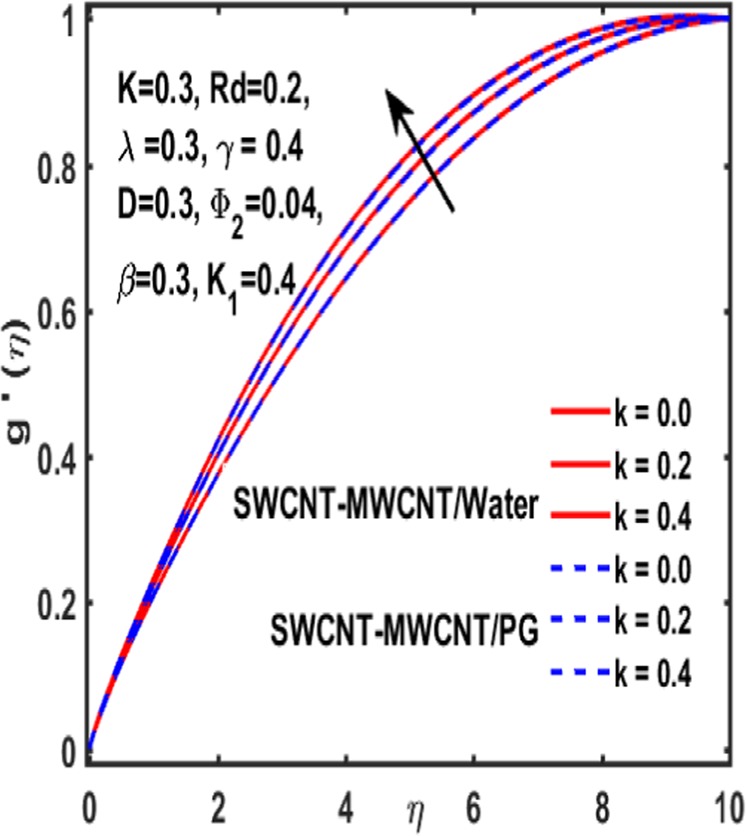
Impact of k on ${\boldsymbol{g}}{\boldsymbol{{\prime} }}({\boldsymbol{\eta }})$.

Here the implications of various parameters like curvature parameter $k$, magnetic parameter $\beta$, reciprocal magnetic Prandtl number $\lambda$, CNTs volumetric fraction for hybrid fluid ${\phi }_{2}$, dimensionless parameter $\gamma$ and material parameter ${K}_{1}$ on induced magnetic field are examined and reported through Figs. 19–23. Consequence of increasing dimensionless parameter $\gamma$ is depicted in Fig. 19. It is revealed that induced magnetic field increases as value of $\gamma$ upsurges. Figure 20 is plotted to demonstrate the impact of material parameter ${K}_{1}$ on magnetic field which shows that a induced magnetic field is escalated with the increasing value of ${K}_{1}\,$. in order to determine the influence of volumetric fraction ${\Phi }_{2}$ on $g{\prime} (\eta )$ Fig. 21 is drawn. This figure illustrates that enhancing the value of ${\Phi }_{2}$ lead to a declining behavoiur of induced magnetic field i.e. curviness decreases. Fig. 22 depicts the variation of $\lambda$ on induced magnetic field. It is reported that induced magnetic field curve mounts drastically as value of $\lambda$ grows. Finally, the influence of curvature parameter $k$ on $g{\prime} (\eta )$ is shown in Fig. 23. It describes that a sharp uplift is sight in curviness of induced magnetic field as the value of $k$ is mounted.

## Conclusions

The present study was conducted to analyze the role of induced magnetic field on microolar hybrid nanofluid flowing towards a stretching curved surface. Single and multiwall CNTs are considered as nanoparticles whereas water and propylene glycol are taken as base fluids. Bvp4c method is selected for mathematical computation of equation obtained from similarity transform. The most convinent and impartant resulst are gained which presented below

- The micropolar rotation increases as volumetric fraction enhances.
- The enhancing the value of ${\Phi }_{2}$ lead to a declining behavoiur of induced magnetic field.
- The micropolar rotation increases as volumetric fraction enhances.
- Increase in value of ${\Phi }_{2}$ display a sharp reduction in $R{e}^{1/2}\,C{f}_{s}$ for water and propylene glycol while $-R{e}^{-1/2}\,N{u}_{s}$ enhancing for both base fluids
